# Effectiveness and Experiences of Quality Improvement Interventions in Older Adult Care: Protocol for a Mixed Methods Systematic Review

**DOI:** 10.2196/56346

**Published:** 2024-04-18

**Authors:** Md Shafiqur Rahman Jabin, Ray Samuriwo, Marcus Chilaka, Emilia Vann Yaroson

**Affiliations:** 1 Department of Medicine and Optometry Linnaeus University Växjö Sweden; 2 Faculty of Health Studies University of Bradford Bradford United Kingdom; 3 School of Health and Social Care Edinburgh Napier University Edinburgh United Kingdom; 4 Logistics, Transport, Operations and Analytics Huddersfield Business School University of Huddersfield Huddersfield United Kingdom

**Keywords:** patient safety, acceptability, accessibility, appropriateness, timeliness, equitability, social care

## Abstract

**Background:**

Quality improvement (QI) interventions are designed to resolve the recurring challenges of care for older individuals, such as working conditions for staff, roles of older individuals in their own care and their families, and relevant stakeholders. Therefore, there is a need to map the impacts of QI interventions in older adult care settings and further improve health and social care systems associated with older adults.

**Objective:**

This review aims to compile and synthesize the best available evidence regarding the effectiveness of policy and practice QI interventions in older adult care. The secondary aim is to understand the care of older individuals and QI intervention-related experiences and perspectives of stakeholders, care providers, older individuals, and their families.

**Methods:**

The mixed methods review will follow the standard methodology used by Joanna Briggs Institute. The published studies will be searched through CINAHL, MEDLINE, PsycINFO, ASSIA, and Web of Science, and the unpublished studies through Mednar, Trove, OCLC WorldCat, and Dissertations and Theses. This review included both qualitative and quantitative analyses of patients undergoing older adult care and any health and care professionals involved in the care delivery for older adults; a broad range of QI interventions, including assistive technologies, effects of training and education, improved reporting, safety programs, and medical devices; the experiences and perspectives of staff and patients; the context of older adult care setting; and a broad range of outcomes, including patient safety. The standard procedure for reporting, that is, PRISMA (Preferred Reporting Items for Systematic Reviews and Meta-Analyses) guidelines, will be followed.

**Results:**

A result-based convergent synthesis design will be used in which both quantitative and qualitative studies will be analyzed separately, and the results of both syntheses will be then integrated during a final (convergent) synthesis. The integration will compare the findings of quantitative and qualitative evidence using tables in light of the results of both syntheses.

**Conclusions:**

This comprehensive review is expected to reflect on the insights into some QI interventions and their impact, outline some common challenges of quality for older adult care, and benefit both the practical usefulness of care service activities and the society at large.

**International Registered Report Identifier (IRRID):**

PRR1-10.2196/56346

## Introduction

### Overview

Quality improvement (QI) intervention has been defined as “a systematic and continuous approach that designs, tests and implements changes using real-time measurements to improve the safety, effectiveness and experience of care” [1]. There are QI interventions designed to illuminate and resolve the current challenges in the care of older individuals that arise within health care and social care systems. This may include the challenges regarding working conditions for staff and the organization as a whole [2]; the roles of older individuals in their own care, including support from their relatives and families [3]; and the roles of clinical and public health researchers, academics, engineers and planning experts [4-7] to make changes in improving health and social care systems for older adults [4,8]. For the readers’ convenience, the definition of QI will be used throughout this protocol to indicate the care of older individuals associated with health and social care systems.

The older population worldwide is growing with increased life expectancy and improving public health. These individuals are expected to live longer with multiple needs and circumstances [9]. Despite the challenge regarding adequate funding, care for older individuals in Sweden has been established because everyone should have equal access to care services [10]. Over 18% of the Swedish population is 65 years or older, and this number is expected to grow and be over 20% by the year 2030 and 25% by 2040 [11]. Sweden’s health and care burden was moved to homecare in 1992 by implementing the ÄDEL reform, which focuses on minimizing complications for older adult care. Even though this strategy turned out to be a success for the Swedish health and social care system, achieving better accessibility by providing home health services to 90% of the aging population [10,12], the challenges around older adult care still remain. For example, a Swedish study found that while government agencies were very positive about the implementation of new technology in older adult care, the unstructured implementation process (without proper planning) and incoherent evaluation model (without compatible designing) indicated inequality of access to such new technology [13,14].

Several QI programs have shown promising results in improving the aging population’s health and social care quality. For example, the QI program, that is, Acute Care for Elders, improved the outcomes for older adults, that is, significant reductions in cost and length of stay with greater comorbidity scores [15]. Other QI interventions, such as education sessions/toolkits, improved the impact of accurate and suitable medicines supply to the residents of residential aged-care facilities [16]. A discharge planning intervention increased the feasibility and effectiveness of facilitating the transition of older adults from hospitals to their homes [17]. An intervention comprising technical and social components reduced preventable harm in care homes [18,19]. Such QI programs may also involve collaboration between geriatricians and primary care physicians, further reducing hospitalization risk and total health and care costs among vulnerable older adults [20].

The review will consider various dimensions of care quality, such as accessibility, appropriateness, safety, efficacy, effectiveness, timeliness, patient-centeredness, and equitability [21,22]. Each subsection will also be covered to obtain a comprehensive insight into health and care quality. For example, the 5 dimensions of accessibility, that is, approachability, acceptability, availability and accommodation, affordability, and appropriateness, will be considered and explored [23,24]. This is because the care provided should be adequate for all these dimensions, that is, 1 or more missing aspects of the care do not complete the entirety of the quality [25,26]. For instance, providing inappropriate or ineffective care should be unacceptable even if it is safe and provided in a timely manner [27,28].

A recent comprehensive or mixed methods systematic review of QI interventions in the radiology setting has demonstrated expected results, that is, improvements in outcomes, such as improved workflow efficiency, report turnaround time, and teamwork and communication [29,30]. We believe that a similar review in the setting of older adult care would develop new knowledge for the health and social care system as well as for older individuals and care providers. Therefore, the findings of the review will generate insights that have wider relevance in creating a model for improved older adult care systems and to meet today’s societal challenges.

Several systematic reviews have been performed for older adult care focusing on health and care quality and its dimensions, such as patient safety [31], appropriateness [32], and accessibility [23], including recent reviews on the interventions of eHealth [33] and training [34]. However, none of these reviews constitutes a comprehensive systematic review focusing on both quantitative and qualitative studies for looking through multiple lenses into the health and social care system of older adult care with broader perspectives that would further facilitate analyzing the issues and devising recommendations. A preliminary search has been performed on Campbell Systematic Reviews, the Cochrane Database of Systematic Reviews, PROSPERO (International Prospective Register of Systematic Reviews), and Joanna Briggs Institute (JBI) evidence synthesis, and we have not identified any systematic reviews on QI interventions in older adult care settings using either qualitative or quantitative methodology.

### Aim and Review Questions

The primary purpose of this review is to compile and synthesize the best available evidence regarding the effectiveness of QI interventions in older adult care. Provided that QI initiatives ultimately target the beliefs and perspectives of clinicians, in a broader sense, the organization and its resources, the secondary objective of this review is to understand the experiences and perspectives of care providers, older individuals and their families, and relevant stakeholders undergoing a QI initiative. Specifically, the review questions are as follows:

- What kind of QI interventions have been used to improve the quality of older adult care?
- How effective are interventions in relation to policy and practice that target improvements in the quality of older adult care?
- What are the experiences and perspectives of care providers, older individuals and their families, and relevant stakeholders about these interventions?

## Methods

The proposed scoping review will be conducted in accordance with the JBI methodology for systematic (mixed methods) reviews [35].

### Search Strategy

Databases will be searched for both published and unpublished studies. The approach to searching for studies for a scoping review will follow the standard 3-step method. The first step will be an initial limited search of a selection of relevant databases, followed by an analysis of text words in the title and abstract and the index terms used to describe the article. The search for published studies will include a 2-way search strategy. One is to search the journal and reference databases, such as CINAHL, MEDLINE, PsycINFO, ASSIA, and Web of Science. Another is to search article-based (journal) databases, such as ACM digital library, IEEE Xplore, and BMJ Journals. The search for unpublished studies (gray literature) will include Mednar, Trove, OCLC WorldCat, and Dissertations and Theses. A second search using all identified keywords (see Textbox 1) and index terms will be undertaken across all included databases. Additional search strategies, that is, citation search—specific researcher or article (for example, gold-standard article), and chain search—review reference list of the systematically selected articles will be included to complement the search for published and unpublished papers. Studies, such as reviews (systematic, scoping, umbrella) and editor letters, will be excluded. Any studies that lack ethical concerns will also be excluded.

**A list of keywords for search strategy.**

**Participants**: Old people (patient), older people (patient), elderly people (patient), elderly people (patient), elderlies, aging population, geriatricians, social workers, and domiciliary workers (Using OR Boolean operator).

Context: elderly care, aged care, primary care, private hospital, public hospital, clinic, geriatric ward, old-age home, nursing homes, domiciliary care, and home care (Using OR Boolean operator).

**Interventions:** Technology, training, education, staff arrangement, incident reporting, peer review, clinical audit, teamwork intervention, communication intervention, team training, safety checklist, local governance, and quality improvement intervention. (Using OR Boolean operator).

**Outcomes:** Patient safety, incident, event, near miss, adverse event, safety culture, safety effectiveness, timeliness, patient-centeredness, equitability, decision-making, communication, teamwork, leadership, report turnaround, patient experience, patient perspective, staff experience, and staff perspective. (Using OR Boolean operator).

**Types of Studies:** Randomized controlled trials, cluster randomized controlled trials, quasi-experimental, controlled before and after trials, interrupted time series analysis, qualitative, grounded theory, ethnography, phenomenology, case study, narrative model, and historical model (Using OR Boolean operator).

Studies published in English will be considered. Studies published from 1990 onward (when the first substantive patient safety research study, “Harvard Medical Practice,” was published) [36,37] will be considered for inclusion in this review. The search strategy results will be depicted through the PRISMA (Preferred Reporting Items for Systematic Reviews and Meta-Analyses) flow diagram [38].

### Inclusion Criteria

#### Research Background

The inclusion criteria of this review will follow the mnemonics of PICO (for quantitative studies) and PICo (for qualitative studies)—which stands for Population, Phenomena of Interest, and Context. These mnemonics are used as a guide (not policy); therefore, the inclusion criteria of this systematic review will include a detailed description of types of participants/population, types of interventions, phenomena of interest, context, outcomes, as well as types of studies, search strategies, assessment of methodological quality, and synthesis of results.

#### Types of Participants/Population

This review will include studies of older individuals (65 years or older) undergoing older adult care irrespective of gender and diversity, including age, ethnicity, socioeconomic status, and disability, and any health and care professionals (care providers) and stakeholders involved in the care delivery for older individuals, such as nurse practitioners, registered nurses, enrolled nurses, specialist nurses, and geriatricians.

#### Types of Interventions

The included studies should concentrate on the implementation of a QI intervention, that is, defined as the systematic and continuous approach that designs, tests, and implements changes using real-time measurements to improve the safety, effectiveness, and experience of older adult care. Given the complexities of the health and social care system, a range of QI interventions will be included in this review. The interventions will include but not are limited to ton technology; training and education; improved reporting and management; safety programs; changes in staffing arrangements (staffing levels, and skill, grade and qualification mix); improved regulation; peer review; and revalidation, clinical audit, and changes to local governance.

#### Phenomena of Interest

The phenomena of interest will be the effects on outcomes and workflow processes and the experiences and perspectives of care providers and older individuals undergoing or being exposed to the QI interventions. These experiences or perspectives included descriptions of the quality and safety concerns, the contexts and cultures of the workplaces (including factors such as conflict and how it was managed, teamwork behaviors, and the attitudes of care providers), and the management of adverse events and near misses.

#### Context

The systematic review will consider studies in older adult care settings, such as geriatric wards of primary health care, hospitals or clinics, old-age homes, nursing homes, and home care facilities for older people.

#### Outcomes

The outcomes will include validated measures of safety culture decision-making, communication, teamwork, leadership, report turnaround time, and timeliness of care. Further outcomes will be patient satisfaction and patients’ perceptions of the quality of older adult care [39].

#### Types of Studies

Quantitative and qualitative studies will be included to report empirical evidence and the human experience. Quantitative studies include randomized controlled trials or Cluster Randomized Controlled trials; nonrandomized controlled trials; quasi-experimental, controlled before-after trials; and interrupted time series studies. Qualitative studies included interpretive work focusing on, but not limited to, designs such as content analysis, phenomenology, grounded theory, ethnography, case study, narrative model, and historical model. Mixed methods and descriptive studies will also be eligible for inclusion.

#### Assessment of Methodological Quality

Two independent reviewers for methodological validity prior to inclusion will assess selected quantitative and qualitative papers for retrieval, reducing the risk of methodological issues, such as subjective bias. This will be done using the standardized critical appraisal criteria from the critical appraisal instruments by the JBI. Any discrepancies that may arise between reviewers’ assessments will be resolved by consensus through discussion.

A critical appraisal of the selected papers will be conducted after screening. The formal process of GRADE (Grading of Recommendations Assessment, Development, and Evaluation) and the GRADE-CERQual (Confidence in the Evidence from Reviews of Qualitative Research) will be used to rate the quality of scientific evidence and confidence to place in findings from systematic reviews of both quantitative and qualitative studies, respectively. For example, 3 grades of quality may be used for each study, based on the score achieved in the critical appraisals: low quality (less than 33% or equal to 33%), medium quality (34%-66%), and high quality (more than 66%).

### Data Extraction

Quantitative data will be extracted from papers included in the review using the standardized JBI data extraction tool. Qualitative data will be extracted from papers included in the review using the standardized JBI data extraction tool. The data extracted for quantitative and qualitative studies will include specific details about the interventions, populations, study methods, and outcomes of significance to the review question and specific objectives.

## Results

A result-based convergent synthesis design [40] will be used in which quantitative and qualitative studies will be analyzed separately, and the results of both syntheses will be then integrated during a final (convergent) synthesis. The integration will compare the quantitative and qualitative evidence findings using tables in light of the results of both syntheses. This will help us address overall review questions with subquestions (if possible).

Quantitative papers will, where possible, be pooled in a statistical meta-analysis using the appropriate JBI tool. All results will be subject to double data entry. Effect sizes expressed as odds ratio (for categorical data) and weighted mean differences (for continuous data) and their 95% CIs will be calculated for analysis. Heterogeneity will be assessed statistically using the standard chi-square and I-square. It will also be explored using subgroup analyses based on the different quantitative study designs included in this review. Where statistical pooling is impossible, the findings will be presented in narrative form, including tables and figures, to aid in data presentation where appropriate.

Qualitative research findings will, where possible, be pooled using the appropriate JBI tool. This will involve the aggregation or synthesis of findings to generate a set of statements that represent that aggregation through assembling the findings rated according to their quality and categorizing them based on similarity in meaning. These categories are then subjected to a meta-synthesis to produce a single comprehensive set of synthesized findings that can be used as a basis for evidence-based practice. Where textual pooling is impossible, the findings will be presented in narrative form.

## Discussion

### Principal Findings

Health and social care systems embrace welfare broadly and include institutions, organizations, and resources to maintain or promote health and social care. The resources may consist of funds for the training and education of care providers and welfare technologies used for older adult care services [41,42]. During the COVID-19 pandemic, the quality and safety of care for older individuals have been a top priority [43]. A future pandemic situation can be overcome through sustainable health and social care systems for older individuals by dealing with the challenges found at the system and organizational levels.

This review is designed to be a mixed methods systematic review that will contain evidence of and bring together the results of single-method reviews (including quantitative and qualitative), which has the potential to produce reviews of direct relevance to policy makers and practitioners. As this review covers a wide range of interventions and outcomes and the experiences and perspectives of both care providers and older individuals, the results are expected to reflect on and offer insights into some QI interventions and their impact and some common quality challenges for older adult care. The challenges may include accommodation and availability, the role of the families, friends, and relatives; working conditions and staffing arrangements; and the practical usefulness of the patients and care service activities and the society at large.

It is essential to cover a diversity perspective in the review; for example, older individuals of different cultural backgrounds may have different needs for QI. As we cover as many health and care quality dimensions as possible, we believe that issues of diverse perspectives will be included in the literature. For example, the dimension of “acceptability” is defined as the relationship between clients’ attitudes about personal and practice characteristics of existing providers, including age, sex, location, and type of facility or religious affiliation of the provider or facility, as well as provider attitudes about acceptable personal characteristics of clients, including ethnicity and source of payment [23,24,44].

This review will include studies of individuals undergoing older adult care irrespective of gender, diversity, ethnicity, socioeconomic status, and disability. This review will evaluate if older adult care facilities worldwide are equipped enough to deal with the challenges of gender sensitivity and cultural diversity. For example, the review may cover the issues of gender sensitivity, especially when a family experiences feelings of shame when receiving care from a nonfamily member or the opposite gender [23,45].

### Strengths and Limitations of the Review

The results of this systematic review will need to be treated with caution since our search is restricted to language and publication period. To minimize the effect of such limitations, a comprehensive strategy, that is, a standard 3-step method, will be followed. For example, the inclusion of gray literature will provide additional insights into the review findings. There may be other limitations, for instance, the possibility pertaining to the results associated with limited included studies, introducing a layer of bias for the selected studies. The evidence-based practice center methods guide proposed by the Agency for Health Care Research and Quality will be used to risk the risk of bias in individual studies [46].

To ensure the review produces generalizable findings, discussions with older adults and their families and the relevant public will be channeled to support the interpretation and dissemination of the review findings.

### Conclusion

No current or underway systematic reviews on the topic were identified. This comprehensive review will uncover the shreds of evidence requiring diligent attention to address the effectiveness of interventions in relation to policy and practice in older adult care settings. The review will also reflect on the insights into the impact of QI interventions, outline some common challenges of quality for older adult care, and benefit both the practical usefulness of care service activities and the society at large.

## Abbreviations

CERQual: Confidence in the Evidence from Reviews of Qualitative Research
GRADE: Grading of Recommendations Assessment, Development and Evaluation
JBI: Joanna Briggs Institute
PRISMA: Preferred Reporting Items for Systematic Reviews and Meta-Analyses
PROSPERO: International Prospective Register of Systematic Reviews
QI: quality improvement

## Data Availability

All data generated or analyzed during this study will be included in this published article and its supplementary information files.

## References

[1] (2016). Quality improvement-training for better outcomes. Academy of Medical Royal Collages.

[2] Ede L, Rantakeisu U (2015). Managing organized insecurity: the consequences for care workers of deregulated working conditions in elderly care. Nord J Work Life Stud.

[3] Tinetti ME, Naik AD, Dindo L, Costello DM, Esterson J, Geda M, Rosen J, Hernandez-Bigos K, Smith CD, Ouellet GM, Kang G, Lee Y, Blaum C (2019). Association of patient priorities-aligned decision-making with patient outcomes and ambulatory health care burden among older adults with multiple chronic conditions: a nonrandomized clinical trial. JAMA Intern Med.

[4] Alavi K, Sutan R, Shahar S, Manaf MRA, Jaafar MH, Maulud KNA, Embong Z, Keliwon KB, Markom R (2022). Connecting the dots between social care and healthcare for the sustainability development of older adult in Asia: a scoping review. Sustainability.

[5] Jabin MSR, Nilsson E, Nilsson A, Bergman P, Jokela P (2022). Digital health testbeds in Sweden: an exploratory study. Digit Health.

[6] Jabin MSR, Pan D (2023). Software-related challenges in Swedish healthcare through the lens of incident reports: a desktop study. Digit Health.

[7] Jabin MSR (2024). Operational disruption in healthcare associated with software functionality issue due to software security patching: a case report. Front Digit Health.

[8] Jabin MSR, Steen M, Wepa D, Bergman P (2023). Assessing the healthcare quality issues for digital incident reporting in Sweden: incident reports analysis. Digit Health.

[9] Fors S, Wastesson JW, Morin L (2021). Growing income-based inequalities in old-age life expectancy in Sweden, 2006-2015. Demography.

[10] (2020). Healthcare in Sweden. Sweden Sverige.

[11] Lennartsson C, Heimerson I (2012). Elderly people's health: health in Sweden: the national public health report 2012. chapter 5. Scand J Public Health.

[12] (2014). Sweden: a role model for elderly care. Global Health Aging.

[13] Frennert S, Baudin K (2021). The concept of welfare technology in Swedish municipal eldercare. Disabil Rehabil.

[14] Tapuria A, Porat T, Kalra D, Dsouza G, Xiaohui S, Curcin V (2021). Impact of patient access to their electronic health record: systematic review. Inform Health Soc Care.

[15] Brennan MJ, Knee AB, Leahy EJ, Ehresman MJ, Courtney HA, Coffelt P, Stefan MS (2019). An acute care for elders quality improvement program for complex, high-cost patients yields savings for the system. J Hosp Med.

[16] Gilmartin JFM, Marriott JL, Hussainy SY (2016). Improving Australian care home medicine supply services: evaluation of a quality improvement intervention. Australas J Ageing.

[17] Dedhia P, Kravet S, Bulger J, Hinson T, Sridharan A, Kolodner K, Wright S, Howell E (2009). A quality improvement intervention to facilitate the transition of older adults from three hospitals back to their homes. J Am Geriatr Soc.

[18] Marshall M, Pfeifer N, de Silva D, Wei L, Anderson J, Cruickshank L, Attreed-James K, Shand J (2018). An evaluation of a safety improvement intervention in care homes in England: a participatory qualitative study. J R Soc Med.

[19] Marasinghe KM, Chaurasia A, Adil M, Liu QY, Nur TI, Oremus M (2022). The impact of assistive devices on community-dwelling older adults and their informal caregivers: a systematic review. BMC Geriatr.

[20] Fenton JJ, Levine MD, Mahoney LD, Heagerty PJ, Wagner EH (2006). Bringing geriatricians to the front lines: evaluation of a quality improvement intervention in primary care. J Am Board Fam Med.

[21] (2006). Quality of Care: A Process for Making Strategic Choices in Health Systems.

[22] King A (2003). Improving quality (IQ): a systems approach for the New Zealand health and disability sector. N Z Med J.

[23] van Gaans D, Dent E (2018). Issues of accessibility to health services by older Australians: a review. Public Health Rev.

[24] Penchansky R, Thomas JW (1981). The concept of access: definition and relationship to consumer satisfaction. Med Care.

[25] Jabin MSR, Magrabi F, Hibbert P, Schultz T, Runciman W (2019). Identifying clusters and themes from incidents related to health information technology in medical imaging as a basis for improvements in practice.

[26] Jabin MSR, Pan D, Nilsson E (2024). Characterizing patient details-related challenges from health information technology-related incident reports from Swedish healthcare. Front Digit Health.

[27] Jabin MSR, Mandel C, Schultz T, Hibbert P, Magrabi F, Runciman W (2019). Identifying and characterizing the 18 steps of medical imaging process workflow as a basis for targeting improvements in clinical practice.

[28] Jabin MSR (2019). Identifying and characterising problems arising from interactions between medical imaging and health information technology as a basis for improvements in practice. Australian Institute for Precision Health.

[29] Jabin MSR, Schultz T, Mandel C, Bessen T, Hibbert P, Wiles L, Runciman W (2022). A mixed-methods systematic review of the effectiveness and experiences of quality improvement interventions in radiology. J Patient Saf.

[30] Jabin SR, Schultz T, Hibbert P, Mandel C, Runciman W (2016). Effectiveness of quality improvement interventions for patient safety in radiology: a systematic review protocol. JBI Database System Rev Implement Rep.

[31] Long SJ, Brown KF, Ames D, Vincent C (2013). What is known about adverse events in older medical hospital inpatients? a systematic review of the literature. Int J Qual Health Care.

[32] Niehoff KM, Mecca MC, Fried TR (2019). Medication appropriateness criteria for older adults: a narrative review of criteria and supporting studies. Ther Adv Drug Saf.

[33] Buyl R, Beogo I, Fobelets M, Deletroz C, Van Landuyt P, Dequanter S, Gorus E, Bourbonnais A, Giguère A, Lechasseur K, Gagnon M (2020). e-Health interventions for healthy aging: a systematic review. Syst Rev.

[34] Lopez P, Pinto RS, Radaelli R, Rech A, Grazioli R, Izquierdo M, Cadore EL (2018). Benefits of resistance training in physically frail elderly: a systematic review. Aging Clin Exp Res.

[35] Peters MDJ, Godfrey CM, Khalil H, McInerney P, Parker D, Soares CB (2015). Guidance for conducting systematic scoping reviews. Int J Evid Based Healthc.

[36] Leape LL, Brennan TA, Laird N, Lawthers AG, Localio AR, Barnes BA, Hebert L, Newhouse JP, Weiler PC, Hiatt H (1991). The nature of adverse events in hospitalized patients. results of the Harvard medical practice study II. N Engl J Med.

[37] Brennan TA, Leape LL, Laird NM, Hebert L, Localio AR, Lawthers AG, Newhouse JP, Weiler PC, Hiatt HH (1991). Incidence of adverse events and negligence in hospitalized patients. results of the Harvard Medical Practice Study I. N Engl J Med.

[38] Page MJ, McKenzie JE, Bossuyt PM, Boutron I, Hoffmann TC, Mulrow CD, Shamseer L, Tetzlaff JM, Akl EA, Brennan SE, Chou R, Glanville J, Grimshaw JM, Hróbjartsson A, Lalu MM, Li T, Loder EW, Mayo-Wilson E, McDonald S, McGuinness LA, Stewart LA, Thomas J, Tricco AC, Welch VA, Whiting P, Moher D (2021). The PRISMA 2020 statement: an updated guideline for reporting systematic reviews. Int J Surg.

[39] Owens DJ, Batchelor C (1996). Patient satisfaction and the elderly. Soc Sci Med.

[40] Hong QN, Pluye P, Bujold M, Wassef M (2017). Convergent and sequential synthesis designs: implications for conducting and reporting systematic reviews of qualitative and quantitative evidence. Syst Rev.

[41] Calnan M, Dingwall R, Quah SR, Cockerham WC (2014). Health and welfare systems. The Wiley Blackwell Encyclopedia of Health, Illness, Behavior, and Society.

[42] Marasinghe KM (2016). Assistive technologies in reducing caregiver burden among informal caregivers of older adults: a systematic review. Disabil Rehabil Assist Technol.

[43] Ministry of Health and Social Affairs (2020). About the COVID-19 virus: for older people, people with health conditions and health and social care staff. Government Offices of Sweden.

[44] Perski O, Short CE (2021). Acceptability of digital health interventions: embracing the complexity. Transl Behav Med.

[45] Iriarte EG, McConkey R, Vilda D (2021). Family experiences of personalised accommodation and support for people with intellectual disability. J Intellect Disabil.

[46] Viswanathan M, Patnode CD, Berkman ND, Bass EB, Chang S, Hartling L, Murad MH, Treadwell JR, Kane RL (2012). Assessing the risk of bias of individual studies in systematic reviews of health care interventions. Methods Guide for Effectiveness and Comparative Effectiveness Reviews.

